# 5-ALA Fluorescence Image Guided Resection of Glioblastoma Multiforme: A Meta-Analysis of the Literature

**DOI:** 10.3390/ijms160510443

**Published:** 2015-05-07

**Authors:** Samy Eljamel

**Affiliations:** Neurological Surgery, High Tech Neuro & Micro Surgery, Edinburgh EH3 8JB, UK; E-Mail: professer.ms.eljamel@gmail.com

**Keywords:** ALA, FIGS, FIGR, glioma, glioblastoma, brain tumor, surgery

## Abstract

Background: Glioblastoma multiforme (GBM) is one of the most deadly cancers in humans. Despite recent advances in anti-cancer therapies, most patients with GBM die from local disease progression. Fluorescence image guided surgical resection (FIGR) was recently advocated to enhance local control of GBM. This is meta-analyses of 5-aminolevulinic (5-ALA) induced FIGR. Materials: Review of the literature produced 503 potential publications; only 20 of these fulfilled the inclusion criteria of this analysis, including a total of 565 patients treated with 5-ALA-FIGR reporting on its outcomes and 800 histological samples reporting 5-ALA-FIGR sensitivity and specificity. Results: The mean gross total resection (GTR) rate was 75.4% (95% CI: 67.4–83.5, *p* < 0.001). The mean time to tumor progression (TTP) was 8.1 months (95% CI: 4.7–12, *p* < 0.001). The mean overall survival gain reported was 6.2 months (95% CI: −1–13, *p* < 0.001). The specificity was 88.9% (95% CI: 83.9–93.9, *p* < 0.001) and the sensitivity was 82.6% (95% CI: 73.9–91.9, *p* < 0.001). Conclusion: 5-ALA-FIGR in GBM is highly sensitive and specific, and imparts significant benefits to patients in terms of improved GTR and TTP.

## 1. Introduction

Glioblastoma multiforme (GBM) is a locally invasive malignant brain cancer that evaded and eluded cure because of its resistance to current surgical, chemotherapeutic and radiotherapeutic techniques. The best that can be offered to patients today is 14 months median survival with maximum safe surgical resection and concurrent chemoradiotherapy using Temozolomide and external beam radiotherapy [1]. To date, almost all patients have died from local brain recurrence and more than 80% of recurrences developed within 2 cm of the resection margin. Hence, the reason for failure of current therapeutic techniques seems more likely than not due to their failure to impart radical local disease control [2,3].

One of the most innovative techniques that came to the forefront in recent years to improve the local control rate in GBM surgery was the use of 5-aminolevulinic acid (5-ALA)-induced fluorescence to guide surgery (FIGR). 5-ALA induces fluorescence due to accumulation of protoporphyrin IX (PpIX) in the cells of GBM leading to intraoperative biological tagging of these cells, the operating surgeon can see, seek and resect easily without increasing collateral brain damage [3,4,5,6,7,8,9,10,11,12,13,14,15,16,17,18,19,20,21,22,23,24,25,26]. A randomized controlled trial (RCT) demonstrated FIGR had doubled the number of patients who had total resection of the enhancing component of their GBM and significantly prolonged their tumor-free survival [5]. FIGR was approved in the European Union and its use expanded worldwide. It was envisaged that FIGR would undergo the same stages of implementation as any other newly introduced surgical technique. Initially there would be two camps: the skeptics who would not use the technology because of doubts mainly self-doubt about their ability to adopt and use the technology effectively and fear that they will loose some business if they cannot adopt the new technology. The second groups of surgeons includes those who are eager to adopt new technology to improve their work and takes it to the next level and drum some new business. The skeptics will use every opportunity to criticize any presentation or publication and introduce hurdles and excuses to slow down adoption of the new technology. Once most surgeons adopt a new technology, there would be an initial precaution to use it selectively because of the steep learning curve. Once they are comfortable with the new technology, there would be enthusiasm to use the technology widely, leading to less selectivity and expansion of indications in the grey area. As a result of this wider usage surgeons start to experience unwanted outcomes, which leads to refining the indications for using the new technology and the introduction of stringent selection of patients. FIGR has been in use for over two decades, hence it is time to perform meta-analyses of the published literature to find out if the initial results of FIGR had been replicated and whether FIGR achieves the desired outcomes when widely adopted by surgeons outside the stringent criteria and scrutiny of an RCT protocol.

## 2. Results

**Figure 1:**
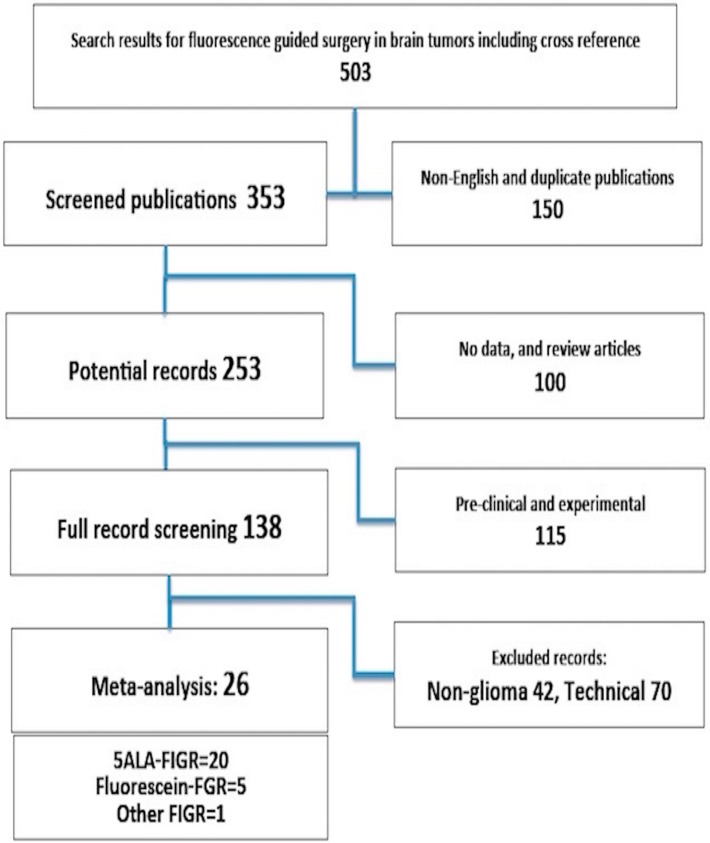
Details of the literature search.

Out of a potentially relevant 26 studies, five reported the use of fluorescein-induced fluorescence image guided resection and one reported on mTHPC (Foscan, Biolitec, Dublin, Ireland)-induced fluorescence image guided surgery (Figure 1). Therefore, only 20 fulfilled all inclusion criteria for this meta-analysis. One study by Eljamel *et al.* 2008 [15] had Photoferin repetitive photodynamic therapy (rPDT) in addition to 5-ALA-FIGR, it was included in the gross total resection (GTR) part of the analysis as rPDT would not had an effect on GTR. The rest of outcome measures it the analyses were performed with this study in and out for completeness as it would not be possible to discern whether 5-ALA-FIGR or rPDT had the major effect. Eight studies were used in meta-analyses of sensitivity and specificity and 12 were used in meta-analyses of FIGR-outcomes.

### 2.1. 5-Aminolevulinic Acid (5-ALA)-Induced Fluorescence to Guide Surgery (FIGR) Gross Total Resection (GTR)

**Figure 2:**
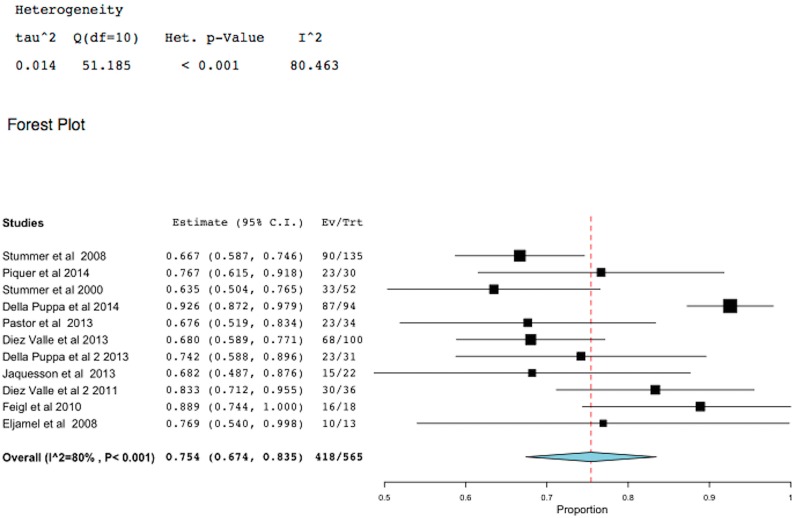
Meta-analysis of gross total resection (GTR) in glioblastoma multiforme (GBM) using 5-aminolevulinic fluorescence image guided surgical resection (5-ALA-FIGR). (Squares represent the mean, the bigger the square is the more weight given because of the narrower 95% CI, the diamond represent the mean in the combined data in the meta-analyses.).

GTR was defined in most studies as absence of contrast enhancement in the resection region of GBM on post-operative T1-weighted MRI scan obtained within the first 72 h after surgery. However, at least one study [11] defined GTR as resection of more than 98% of the enhanced lesion. Eleven studies have reported clearly this outcome; 418/565 (75.4%) had GTR in these studies (95% CI: 67.4–83.5, *p* < 0.001) Figure 2.

### 2.2. 5-ALA-FIGR Time to Tumor Progression

**Figure 3:**
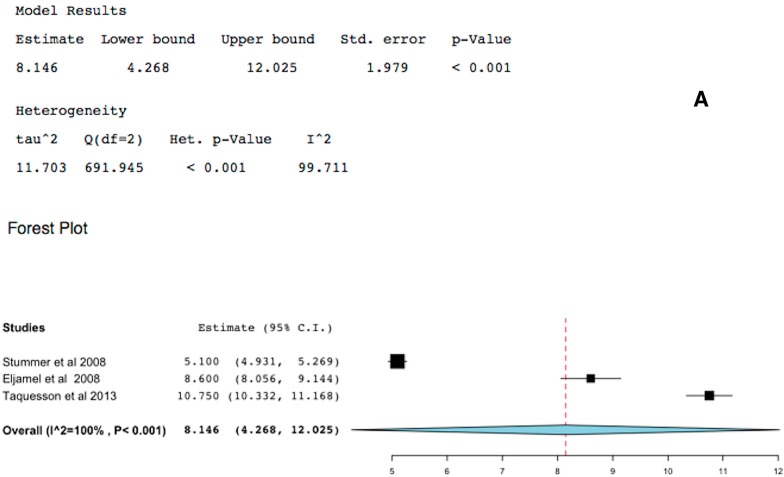

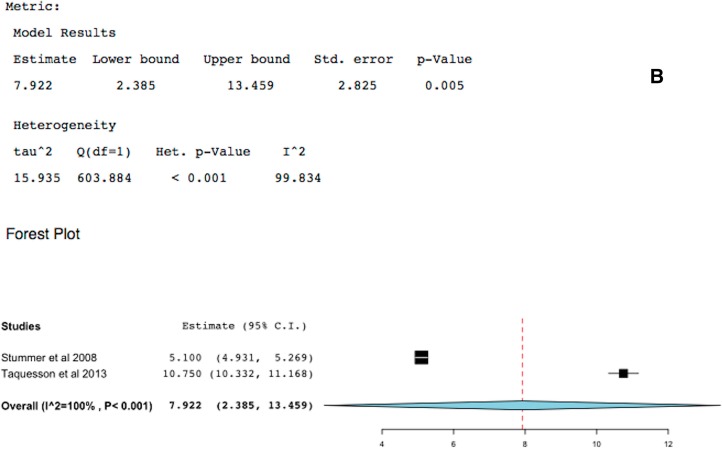
Forest plot (meta-analysis) and heterogeneity of 5-ALA-FIGR time to tumor progression (TTP). (**A**) excludes study [15] and (**B**) includes it, because study [15] had repetitive PDT (rPDT) in addition to 5-ALA-FIGR. (Squares represent the mean, the bigger the square is the more weight given because of the narrower 95% CI, the diamond represent the mean in the combined data in the meta-analyses.).

Time to tumor progression (TTP) should always be considered the primary endpoint of any study of GBM treatment. Three studies reported TTP. One of these studies reported 5-ALA-FIGR and rPDT, therefore the analyses were performed with this study-data in and out. Despite heterogeneity, which was significant (*p* < 0.05), the mean TTP of the collated data was 8 months (95% CI: 4.3–12 months, *p* < 0.001), Figure 3.

### 2.3. 5-ALA-FIGR Survival

**Figure 4:**
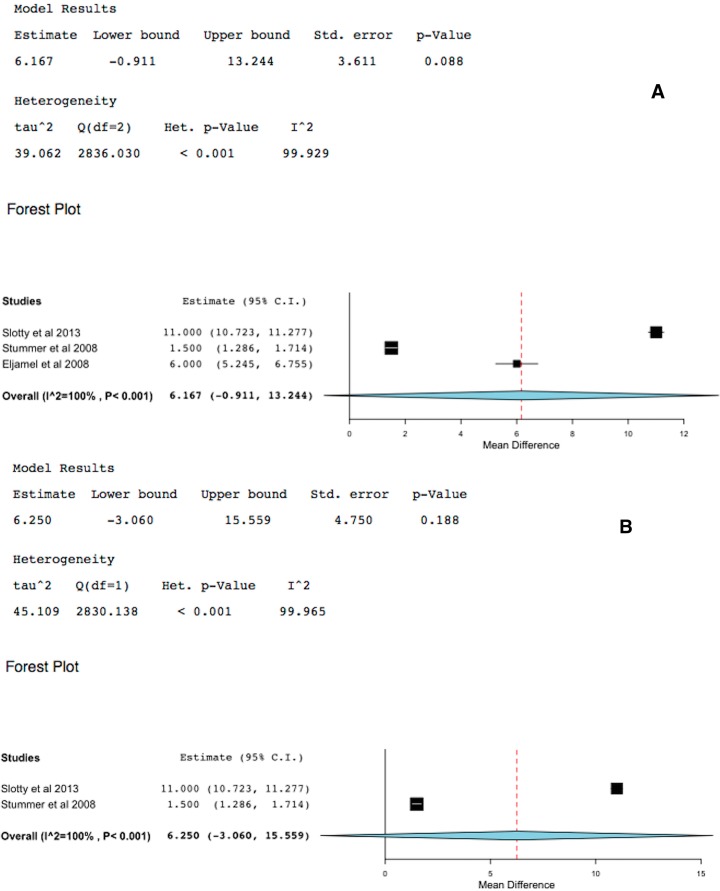
Forest plot (meta-analysis) and heterogeneity of 5-ALA-FIGR survival difference. (**A**) excludes study [15] and (**B**) includes it, because study [15] had rPDT in addition to 5-ALA-FIGR. (Squares represent the mean, the bigger the square is the more weight given because of the narrower 95% CI, the diamond represent the mean in the combined data in the meta-analyses.).

Over all survival in GBM is an inappropriate primary endpoint because patients and surgeons found it difficult to control what other treatments and interventions are carried out after tumor recurrence. Nevertheless, three studies with matched controls (two were RCTs) reported on overall survival of GBM patients treated with 5-ALA-FIGR or not. One of these studies was compounded by the fact that rPDT was used in addition to 5-ALA-FIGR in the study arm [15]; therefore, the analyses were performed both including and excluding this study. The difference in survival in favor of 5-ALA-FIGR was 4–6.2 months (95% CI: −1 to 13, *p* < 0.001), Figure 4. However, the 95% CI was very wide and the effect may well be due to adjuvant therapies, therefore 5-ALA-FIGR alone does not seem to impart significant overall survival benefit on its own or the current studies were underpowered to detect a difference between the study arms.

### 2.4. 5-ALA-FIGR Specificity

**Figure 5:**
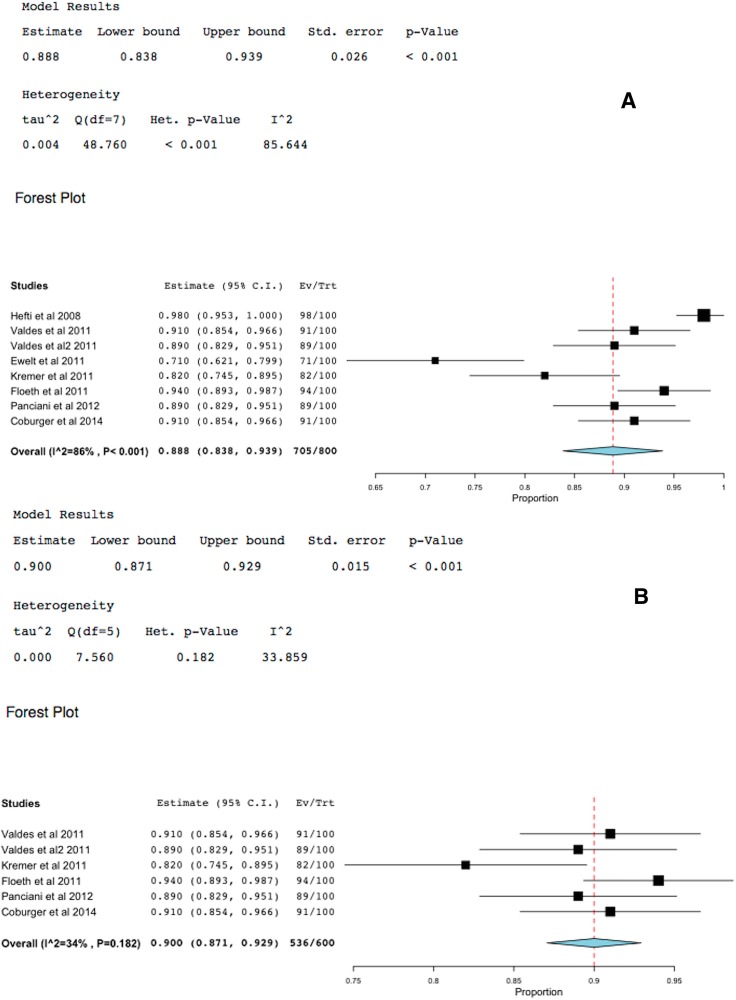
Forest plot (meta-analysis) and heterogeneity of 5-ALA-FIGR specificity. (**A**) excludes studies with non-GBM data and (**B**) includes them, because studies included other types of tumors other than GBM. (Squares represent the mean, the bigger the square is the more weight given because of the narrower 95% CI, the diamond represent the mean in the combined data in the meta**-**analyses.).

Specificity was defined as the percent of specimens that were fluorescence-negative and contained no GBM cells. Eight studies reported 5-ALA-FIGR specificity including 800 histopathological specimens. Two studies included non-GBM data and therefore the analyses were performed with and without these studies. The specificity was 88.8%–90% (95% CI: 83.8%–93.9%, *p* < 0.001), Figure 5.

### 2.5. 5-ALA-FIGR Sensitivity

**Figure 6:**
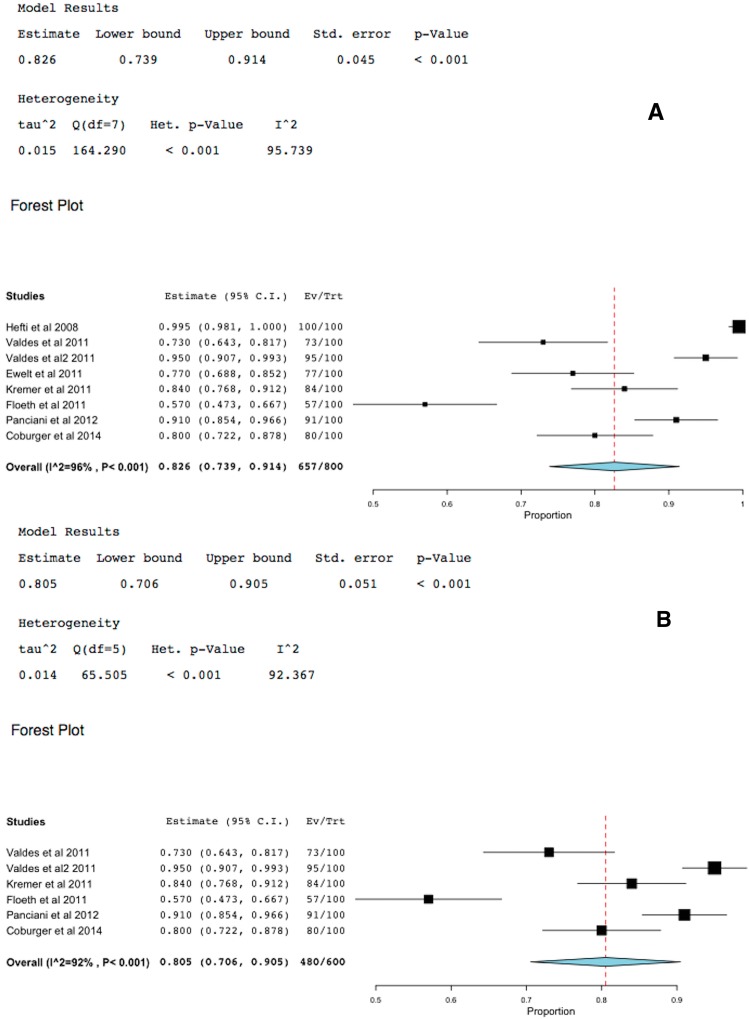
Forest plot (meta-analysis) and heterogeneity of 5-ALA-FIGR sensitivity. (**A**) excludes studies with non-GBM data and (**B**) includes them, because studies [19,22] included other types of tumors other than GBM. (Squares represent the mean, the bigger the square is the more weight given because of the narrower 95% CI, the diamond represent the mean in the combined data in the meta**-**analyses.).

Sensitivity was defined as the percent of specimens of GBM that fluoresced using 5-ALA-FIGR. Eight studies reported on this. Two studies included non-GBM data and therefore the analyses were performed with and without these studies. The sensitivity of 5-ALA-FIGR was 81%–82.6% (95% CI: 73.9%–91.4%, *p* < 0.001), Figure 6.

## 3. Discussion

GTR is an independent prognostic factor for TTP and survival in GBM and a GTR of 98% or more of the enhancing lesion on MRI is considered to be best for GBM surgery [2,3,7]. In the best hands, GTR under white light microsurgery is achieved in about 36% of patients [5] and under the care of non-specialized neurosurgeons more likely to be about 25%. 5-ALA-FIGR improved the GTR in GBM to >64% [2,3,4,5,6] and when compared to white light microsurgery 5-ALA-FIGR was highly significant (*p* < 0.001) [5]. In this meta-analyses that included 11 studies that clearly reported this outcome [4,5,7,8,9,10,11,12,13,14,15]; 418/565 (75.4%) had GTR in these studies (95% CI: 67.4–83.5, *p* < 0.001). Furthermore, it is likely that the extent of surgical resection is much more than post operative MRI scan revealed because several studies had demonstrated that 5-ALA-fluorescence revealed and enabled resection of a much wider lesion than the enhanced MRI scan had revealed, it is estimated that fluorescent GBM extends by 10 mm or more beyond the enhanced edge [16,17]. Therefore, postoperative enhanced MRI scan within the first 72 h is a poor tool to evaluate the true extent of surgical resection in GBM. However, it is the standard of care currently in use.

Time to tumor progression (TTP) should always be considered the primary endpoint of any study evaluating GBM treatment, because in the context of an RCT, it would be difficult and probably un-ethical to control what further therapy or intervention the patient, surgeon or oncologist will choose after GBM recurrences. Three studies included in this meta-analysis reported TTP [5,12,15]. Despite the heterogeneity of these studies, which was significant (*p* < 0.05), the mean TTP with 5-ALA-FIGR was 8.1 months (95% CI: 4.3–12 months, *p* < 0.001). In the two RTCs included in this study, the TTP was significantly longer in the 5-ALA-FIGR arm compared to white microsurgery arm (*p* < 0.001) [5,15].

Though one of these studies was compounded by the addition of Photoferin rPDT [15], repeating the analysis without this study, the TTP was still significant in favor of 5-ALA-FIGR.

Furthermore, patients in the 5-ALA-FIGR had significantly less rescue interventions, such as further surgery, chemotherapy and other interventions [5], resulting in significant healthcare cost-savings and longer quality of life for patients.

Overall survival in GBM is an inappropriate primary endpoint because patients and surgeons found it difficult to control what other treatments and interventions are carried out after tumor recurrence. Nevertheless, three studies with matched controls (two were RTCs) reported on overall survival of GBM patients treated with 5-ALA-FIGR or not [5,15,18]. The difference in survival in favor of 5-ALA-FIGR was 6.2 months (95% CI: −1 to 13, *p* <0.001). However, the 95% confidence interval was very wide and heterogeneity was also significant because of the aforementioned reasons: patients in the control group often received further surgery and rescue therapies that diluted the survival gain of patients who received 5-ALA-FIGR upfront. Though one of these studies was compounded by the addition of Photoferin rPDT [15], repeating the analysis without this study the outcome was still significant in favor of 5-ALA-FIGR. However, because these studies were underpowered and there was no control for rescue and adjuvant therapies, no firm conclusions can be drawn from the published data so far to indicate 5-ALA-FIGR alone imparts significant over all survival advantage.

The current meta-analyses based on eight studies that reported histopathological confirmation of over 800 samples of 5-ALA-fluorescence positive or negative [19,20,21,22,23,24,25,26] demonstrated 5-ALA-FIGR had very high specificity of 88.8% (95% CI: 83.8%–93.9%, *p* < 0.001) and equally high sensitivity of 82.6% (95% CI: 73.9%–91.4%, *p* < 0.001). Excluding two studies that were not pure GBMs did not change materially these results. This high sensitivity and specificity of 5-ALA-FIGR is unmatched by any other intraoperative adjuvants used to enhance the GTR such as image guided surgery (ISG), intraoperative ultrasound (IoUS) or intraoperative MRI (iMRI).

In a previous systematic review of the literature that included 12 studies reported sensitivity of fluorescence guided surgery (FGS) for high-grade glioma of 84% and specificity of 91% and reduced the risk of tumor progression (HR 0.73) [27]. However, the aforementioned review included studies using fluorescein-guided surgery, which were excluded from this meta-analyses [28,29,30,31,32] to eliminate heterogeneity, as the mechanism by which fluorescein and 5-ALA mark GBM cells is entirely different. We have also excluded a further matched controlled study of FIGR, which was not included in the previous systematic review because it reported the use of mTHPC (Foscan) to guide surgery [33]. Furthermore, another systematic review of the literature published two years ago included only 10 studies and concluded ALA-fluorescence guided surgery had a sensitivity of 87%, specificity of 89% [34], which are higher than this meta-analysis because of the stricter selection criteria and this meta-analyses had additional published data. The selective biases that occur as new technology is adopted into main-steam practice are evident by the slide downward of these results as the selection of suitable patients becomes blurred as the experience develops during the learning curve.

It is clear that the extent of surgical resection is an important significant prognostic factor in the TTP and survival in high-grade gliomas and 5-ALA-FIGR is the best available technique to achieve maximum safe surgical resection, which is associated with very few local or systemic side effects [5,35,36]. RCT [5] demonstrated similar complication rates in the 5-ALA-FIGR and the control group. However, the holly grail of eradicating the GBM by extending the surgical resection should not override the basic principle of patient safety and preservation of function as extending surgical resection from >98% excision to total removal of the enhancing lesion resulted in similar outcomes. Furthermore, tumors near eloquent brain areas may require additional techniques such as awake craniotomy to minimize the risk of collateral damage.

## 4. Experimental Section

The medical literature was searched extensively, beginning with basic searches of the MEDLINE/PubMed service of the US National Library of Medicine, using the MeSH (medical subject heading) terms “fluorescence,” “5-aminolevulinic acid (5-ALA),” “glioma,” “brain,” “glioblastoma,” “neurosurgery,” and “surgery” in various combinations. Furthermore, Web of Knowledge database, BIOSIS Previews, Cochrane library, and Web of Science were searched.

Each article of interest was screened and its reference list was checked to make sure that no relevant article was missed. The Internet itself was searched for leads to articles appearing in journals not indexed in these databases.

The relevant clinical studies were selected on the basis of one of the following selection criteria:

(1) Reported sensitivity and specificity of 5-ALA-fluorescence identification of GBM;
(2) Gross total resection of 5-ALA-fluorescence-image guided resection (FIGR) was reported;
(3) The outcomes in terms of time to tumor progression or overall survival were reported.

When the same patient population was used in several publications from the same authors or same institutions, only the most recent study was included in this meta-analysis. Full-text copies of the articles upon which this meta-analysis was based were examined Articles that did not deal with 5-ALA-guided surgery, and non-GBM diagnosis were excluded. Articles that were not in English, contained insufficient data or were technical in nature were also excluded. Only one study was in French was included as it was possible to translate it into English [12].

Eventually, 503 potential articles were accumulated for review (Figure 1). Only 20 out of a potential 26 articles that met the inclusion criteria, were included in the final meta-analyses.

The meta-analyses were conducted according to statistical heterogeneity between the studies using Open MetaAnalyst Software version 0.1 (Brown University, Providence, RI, USA) for Mac. If there was no heterogeneity, fixed effects model was used for meta-analysis; otherwise, a random effect model was used. Statistical heterogeneity was explored by χ2 and inconsistency (I2) statistics; an I2 value of 50% or more represented substantial heterogeneity. Meta-analysis is a statistical technique for combining the findings of two or more independent studies to provide a more precise estimates of treatment effects, giving due weight to the size of the different studies included in the analyses.

## 5. Conclusions

5-ALA-FIGR in GBM is highly sensitive and specific, and imparts significant benefits to patients in terms of improved GTR and TTP. Although the extent of surgical resection is an important prognostic factor in GBMs and 5-ALA-FIGR significantly improved the extent of surgical resection of GBM, at the moment it is not clear that 5-ALA-FIGR alone improves overall survival of GBM. Further RCT with large study sample and appropriate control of adjuvant therapies is warranted.
